# 

**Published:** 1857-05

**Authors:** 


### CONTENTS.

### *ORIGINAL COMMUNICATIONS.*

PAGE

ARTICLE I.—Foreign Correspondence. By W. F. WESTMORELAND, M. D., Professor of the Principles and Practice of Surgery in the Atlanta Med. College, ........ 513

ARTICLE II.—On some of the Medicinal Properties and Therapeutical Applications of Chlorate of Potassa. By J. S. WEATHERLY, M. D., Montgomery Ala., ........ 517

ARTICLE III.—Removal of a piece of Gun Barrel from the outer surface of the Third Rib. By A. W. GRIGGS, M. D., Newnan, Ga., April 10th, 1857, 520

### *SELECTIONS.*

A Monagraph on Ovarian Tumors, ........ 523

The Causes and Prevention of Yellow Fever in New Orleans and other Cities in America, ........ 533

Obstetric Notes and Reflections, ........ 540

On the general use of Chloroform, ........ 543

Cogitations and Vaticinations, ........ 547

Extracts from the Records of the Boston Society foi Medical Improvement, 553

Use of Belladonna in arresting secretions of Milk in swollen and painful Breasts, ........ 558

### *EDITORIAL AND MISCELLANEOUS.*

Atlanta Medical College, ........ 559

"The Little Sister," ........ 565

Controversial character of this No. of the Journal, ........ 568

Circular Savannah Medical College, ........ 569

Proceedings of the Annual Meeting of the Medical Society of the State of Georgia, held at Augusta, April 8th and 9th, 1857, ........ 571

Professor Campbell's Claim, ........ 574

Obstetrical Memoranda, ........ 574

Next Class, ........ 576

